# Mechanical performance and gamma attenuation capability of sustainable pozzolanic cement incorporating basalt under different curing regimes

**DOI:** 10.1038/s41598-025-28919-1

**Published:** 2025-12-07

**Authors:** Ahmed Abd Al-Aziz, F. I. El-Hosiny, Alaa Mohsen, M. Ramadan

**Affiliations:** 1 Chemistry Department, Faculty of Science, Ain Shams University, Cairo, Egypt; 2 Faculty of Engineering, Ain Shams University, Cairo, Egypt

**Keywords:** Blended cement, Basalt, Hydrothermal curing, Mechanical performance, Radiation shielding, Engineering, Environmental sciences, Materials science

## Abstract

This study introduces basalt powder as a novel supplementary cementitious material (SCM) to address the growing scarcity of conventional ones such as silica fume, slag, and fly ash. The research explores the potential of basalt to produce environmentally friendly blended cement with satisfactory mechanical and functional performance. Cement was partially replaced with 10, 20, and 30 wt% basalt to evaluate its influence on fresh properties (workability and setting time), as well as compressive-strength. To assess its suitability for precast applications, basalt-blended cement specimens were subjected to hydrothermal curing at 140, 170, and 200 °C for 3 h, and their compressive-strengths were compared to those obtained under normal curing conditions. Microstructural and phase analyses were conducted using XRD, TGA/DTGA, SEM/EDX, and nitrogen adsorption/desorption techniques. Furthermore, the study investigates the radiation shielding capability of basalt-blended cement against gamma-rays emitted from ^137^Cs (661.64 keV). The results reveal that basalt incorporation reduces workability and prolongs setting time, with 20 wt% basalt identified as the optimal replacement level, achieving a compressive-strength of 69 MPa at 28 days. Hydrothermal curing at 170 °C for 3 h yielded strength comparable to that of normally cured specimens, demonstrating its effectiveness for precast production. Additionally, basalt-enhanced cement showed improved gamma-ray shielding, increasing the linear attenuation coefficient by 11% and reducing the half-value layer by 10%. These findings confirm the dual functionality of basalt as a sustainable SCM and a radiation-shielding additive, especially when combined with hydrothermal curing.

## Introduction

Egypt’s total annual cement production reached approximately 85 million tons, making it one of the world’s largest cement-producing countries, currently ranking 14th globally and first in Africa. Despite significant developments in Egypt’s infrastructure in recent years, the cement industry remains one of the most energy-intensive sectors, consuming non-renewable natural resources and significantly contributing to ecosystem degradation through massive CO_2_ emissions, as well as the release of nitrogen oxides, sulfur oxides, and cement dust^1^.

Key raw materials, such as limestone, gypsum, and clay, are particularly vulnerable due to their intensive and unsustainable consumption in the cement industry, as well as the enormous amount of thermal energy consumed to form clinker^2^. To bring down the depletion of natural resources and energy saving in cement production, several alternative and sustainable solutions can be implemented to mitigate the economic and environmental burdens of this industry, including the utilization of supplementary cementitious materials (SCMs), waste recycling, development of new types of low-carbon cement (geopolymers) and exploitation of alternative fuels such as solid waste and biofuel^3,4^. SCMs are considered green siliceous–aluminous precursors that are presented as byproducts or natural resources. Many construction scientists have focused on the manufacture of pozzolanic cement based on the use of some solid waste, such as slag^5^, fly ash^6^, glass waste^7^, rice husk ash^8^, silica fume^9^, dolomite waste^10^, brick waste^11^, sludge^12^, bypass^13^, marble dust^14^, dealuminated metakaolin^15^ and mineral wool waste^16^. Other researchers have relied on natural SCMs, such as green clays and volcanic rocks, including kaolinite, bentonite, illite, rhyolite, basalt, volcanic tuff, granite, and feldspar minerals^17^.

Basalt is an igneous rock created by the rapid cooling of lava. In general, albite (NaAlSi_3_O_8_), anorthite (CaAl_2_Si_2_O_8_), augite (Ca(Mg, Fe)Si_2_O_6_), and magnetite (Fe_3_O_4_) are the main mineralogical phases of these rocks^18^. The content of silica ranges from 40 to 50% with significant percentages of Al_2_O_3_, CaO, MgO, Fe_2_O_3_ and TiO_2_^19^. In Egypt, major basaltic outcrops are concentrated in the Fayum Depression, the Eastern Desert, and North Sinai. Therefore, many industrial companies in Egypt (CEMEX, Lafarge, Suez, El-Arish Cement Co.) have exploited basalt rocks as a natural pozzolanic material to reduce energy, CO_2_-emissions, heat of hydration and to improve durability^20^.

Basalt powder exhibits reasonable pozzolanicity due to its high silica and alumina content. Additionally, it helps to slow down the rate of heat release during the cementing reactions^21^. Chendaprasert et al. reported that replacing cement with 10–30% basalt powder (BP) along with 10% silica fume (SF) resulted in a marked reduction in early compressive strength; however, strength development was observed over time. At 28–90 days, composites containing 10% silica fume and 10–15% basalt powder exhibited a decrease in porosity, microstructural densification, and improved mechanical performance^22^. Shen et al. concluded that BP and SF can serve as SCMs that enhance the polymerization of calcium silicate hydrate (CSH) networks. However, an excessive amount of BP may cause decalcification of the CSHs structures, leading to a reduction in the strength of cementing composites. These adverse effects, however, can be mitigated by high curing temperatures at 90 °C for 6 h. BP has been utilized by Dobiszewska et al. as a partial cement replacement in mortar mixes, with substitution levels as high as 20%; the study indicated that BP lowers the heat of hydration, slows the hydration process, elongates setting times and leads to a decrease in compressive strength^23^. El-Didamony et al. observed the low pozzolanicity of BP at early hydration ages, but it improved progressively over time^24^. Binici et al. confirmed that grain size has an impact on the functional performance of basalt. Various proportions of basalt, in both powder and sand forms, were incorporated as partial replacements for cement and fine aggregate, respectively. Replacing 10% of the cement with BP and 40% of the fine aggregate with basalt sand resulted in the production of highly durable concrete^25^.

Basalt powder as SCMs provides many economic and environmental benefits, but it also comes with several challenges and limitations that need to be carefully managed, such as low pozzolanic activity at early ages, increased porosity and decalcification at high replacement, which negatively affect the mechanical performance of the cementitious matrix. Hydrothermal treatment presents a promising approach to addressing these challenges. Autoclaving pozzolanic cement at steam pressures and temperatures ranging from 120 to 200 °C significantly accelerates the hydration rate and enhances the pozzolanic reactivity of SCMs^26,27^. This leads to the formation of substantial amounts of highly crystalline and thermally stable hydration products, such as CSH, CASH, and CAH phases^28^. Additionally, hydrothermal curing improves the microstructure by refining the pore structure, reducing overall porosity, minimizing long-term shrinkage, and ultimately enhancing the durability of cementitious composites^29^.

Gamma rays present serious hazards to biological systems because of their strong penetrating power and high ionizing properties^30^. Upon interacting with living tissues, it can cause DNA damage that may result in mutations, cell death, or uncontrolled cell proliferation, potentially leading to cancer^31^. High-dose exposure can lead to acute radiation syndrome, including organ failure or even death^32^. Beyond biological effects, gamma rays can also degrade the performance and reliability of electronic materials and components. Therefore, stringent radiation shielding and safety measures are critical in environments where gamma radiation is prevalent, such as nuclear facilities, medical imaging and therapy units, and space exploration missions^33^.

Although lead (Pb) remains a widely used material for gamma radiation shielding, its application is increasingly limited due to its toxicity, high cost, and substantial weight^34^. Alternative heavyweight aggregates, such as barite^35^, magnetite^36^ and hematite^37^ have demonstrated excellent shielding capabilities owing to their high densities. However, their widespread use is constrained by economic and regional availability challenges. This situation underscores the urgent need to identify and develop cost-effective, sustainable cementitious materials that can provide both adequate radiation protection and strong mechanical performance. Despite the abundance and mineral richness of basalt, its potential as a multifunctional SCM remains underexplored, particularly in the context of radiation shielding applications. Existing studies have primarily focused on its mechanical contributions, with limited attention to its gamma-ray attenuation properties, especially when subjected to thermal activation techniques such as hydrothermal curing. Furthermore, there is a lack of integrated research that simultaneously evaluates mechanical strength, microstructural evolution, and radiation shielding efficiency in basalt-blended cement systems.

Aligned with the goals of Egypt Vision 2030, which emphasize sustainability, circular economy principles, and climate change mitigation. This study aims to fill these gaps by investigating the performance of basalt as an SCM in blended cement, evaluating its effects on fresh and hardened properties, and exploring its potential for precast applications through hydrothermal curing. Additionally, the study uniquely assesses the gamma-ray shielding capability of basalt-blended cement against hazardous isotopes such as ^12^⁷Cs, providing a comprehensive understanding of its multifunctional role in sustainable construction materials.

## Materials and testing methods

### Materials

**Fig. 1:**
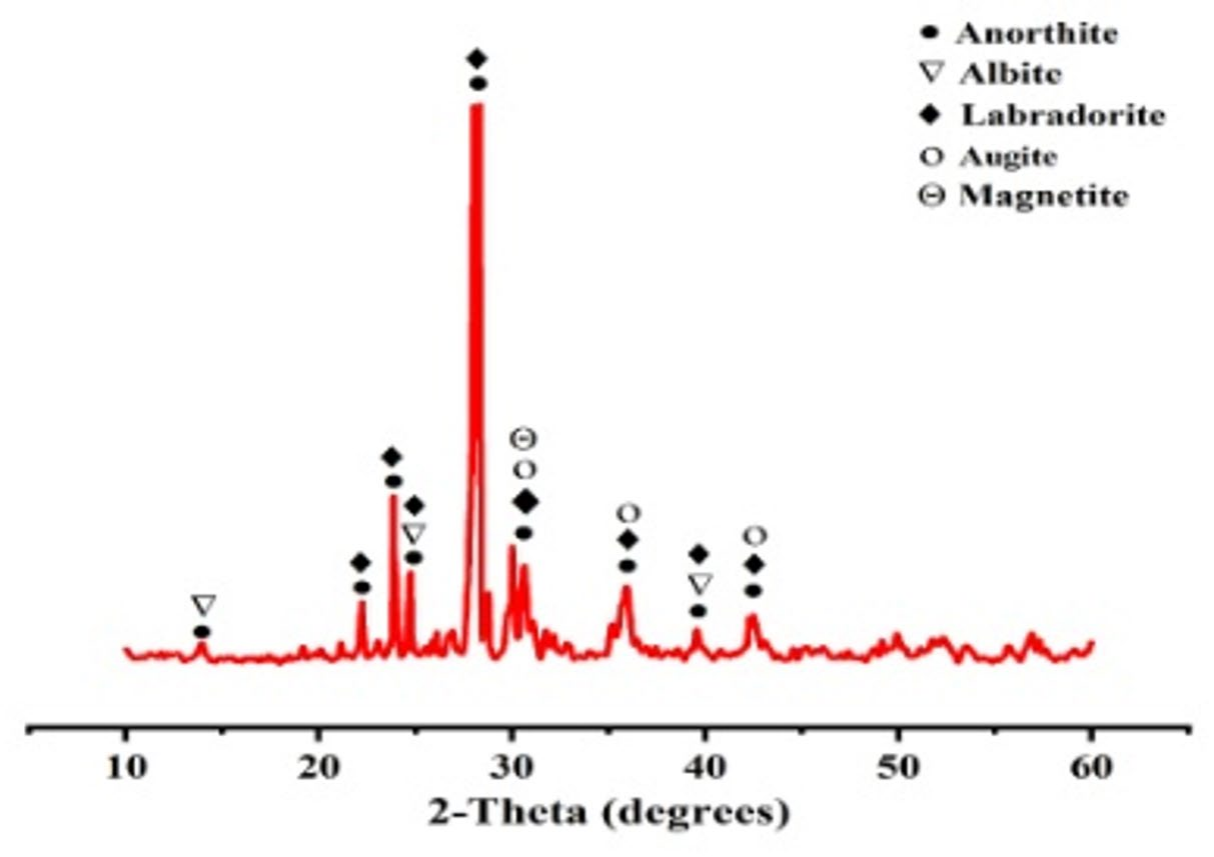
XRD of basalt.

In this study, ordinary-Portland cement (OPC) and basalt were the materials used to prepare blended cement. The OPC with a type CEM I (42.5 N) was delivered from El-Arish Cement Plant, while basalt was obtained from Sinai Cement Company in Egypt. The specific surface-area of OPC and basalt was measured using the Blaine Air-Permeability Apparatus (BAP, model: H-3056.3 F); it was found to be 3320 and 4200 cm^2^/g, respectively. The oxide composition of OPC and basalt was analyzed using X-ray fluorescence (XRF; model: Thermo Scientific ARL 9900), as shown in Table 1. Also, the phase composition of basalt was examined using X-ray diffraction (XRD: model: Malvern-Panalytical, X’Pert-Pro), as represented in Fig. 1. The XRD-pattern of basalt shows the presence of crystalline phases from anorthite (CaAl_2_Si_2_O_8_, PDF# 96-100-0035), albite (NaAlSi_3_O_8_, PDF# 96-900-3703), labradorite (Ca_0.67_Al_1.67_Si_2.33_Na_0.33_O_8_, PDF# 96-210-8234), augite (Ca_0.6_Al_0.34_Si_1.82_Fe_0.2_Mg_0.9_Na_0.1_O_6_, PDF# 96-100-0036) and magnetite (Fe_3_O_4_, PDF# 96-101-1033).

**Table 1:** Oxide compositions for OPC and basalt (mass, %).

Material	SiO_2_	Al_2_O_3_	Fe_2_O_3_	CaO	MgO	SO_3_	Na_2_O	K_2_O	Cl	TiO_2_	LOI
OPC	19.08	5.16	4.07	63.41	1.60	2.91	0.35	0.25	0.00	0.00	4.00
Basalt	48.38	14.30	16.40	11.80	2.85	0.23	1.58	1.18	0.11	2.03	0.31

### Specimens’ preparation and testing

Initially, four dry powders of blended cement were prepared by mixing OPC with different proportions of basalt (0, 10, 20 and 30 wt% with respect to OPC, coded C, C-10B, C-20B and C-30B, respectively) in a ball mill for 1 h, as represented in Table 2. The obtained powders were mixed with a constant amount of water (water/powder (W/P) ratio = 0.26) in a Hobart mixer to prepare fresh cement pastes.

Regarding the fresh properties, the workability of the prepared fresh pastes was evaluated using the mini-slump test. Abram’s cone with a height of 59 ± 1 mm, top diameter of 19 ± 1 mm and bottom diameter of 39 ± 1 mm, placed on a smooth stainless-steel surface was used in this test. The paste was filled into the cone. After that, the cone was removed, leaving the paste to flow, forming a pancake shape. The diameter of the pancake (spreading area) was measured as an indication of workability^38,39^. In the mini-slump test, a high amount of mixing water was used (water/powder ratio = 0.45) to monitor the difference in the spreading area^40,41^. Also, the initial/final-setting time (I/F-ST) was measured using the Vicat apparatus according to ASTM C191-19^42^.

For mechanical performance, the fresh specimens were cast in 1-inch stainless-steel cubic-molds, then cured in a humidity chamber for 24 h at a relative-humidity of 98 ± 2%. The specimens were demolded and divided into groups to study the impact of different curing conditions: (i) normal cured group, under tap water at 25 °C for 1, 28 and 56 days; and (ii) hydrothermal cured group, in an autoclave (model: Control, L0032) at 140, 170 and 200 °C (equivalent to 4, 7 and 10 bar, respectively) for 3 h. At each condition, three samples of each specimen were tested using a compression machine (Control, Max-load of 250 kN), then the average compressive-strength values were calculated.

For some selected specimens, the phase composition of hydration products was examined using XRD and thermogravimetric analysis (TGA/DTG, model: TA-instrument, SDT-Q600). Also, the texture characteristics (specific surface area (m^2^/g), average pore-diameter (nm) and total pore-volume (cm^3^/g)) were analyzed by a surface-area and pore-size distribution analyzer (N_2_-gas adsorption-desorption at 77 K, model: BELSORP^®^ MINI X). Moreover, the morphology of the hydration products and the microstructure of hardened specimens were investigated using a scanning electron microscope connected with Energy-dispersive X-ray (SEM/EDX: model: thermoscientific, Quattro-S).

To investigate the effect of basalt and various curing conditions (normal and hydrothermal curing) on the gamma-radiation shielding efficiency of the fabricated composite, the linear-attention coefficient (µ) and half-value layer (HVL_) were measured against ^137^Cs for some specimens. ^137^Cs was used as a source of gamma-ray with an intensity of 661.64 keV. The specimens were positioned far from the ^137^Cs source by 15 cm; the transmitted gamma-ray intensity was measured by a 3′ × 3′ NaI (TI) detector. Various thicknesses of each specimen (0, 2.5, 5, 7.5, 10, and 12.5 cm) were used to determine the optimal thickness for shielding radiation. The µ and HVL were calculated using Eq. (1) (Beer–Lambert’s law) and (2), respectively.

1 $${\text{I}} = {\text{ I}} \circ {\text{e}}^{{ - \mu {\text{x}}}}$$

2 $${\text{HVL}} = {\text{ ln 2}}/\mu$$

where, I◦ and I: incident and transmitted gamma-ray intensities, respectively; and x: thickness of the specimen.

**Table 2:** Mix design of the prepared pastes (mass, %).

Composite	OPC	Basalt	W/*P* ratio
C	100	–	0.26
C-10B	90	10	0.26
C-20B	80	20	0.26
C-30B	70	30	0.26

## Results and discussions

### Fresh properties

**Fig. 2:**
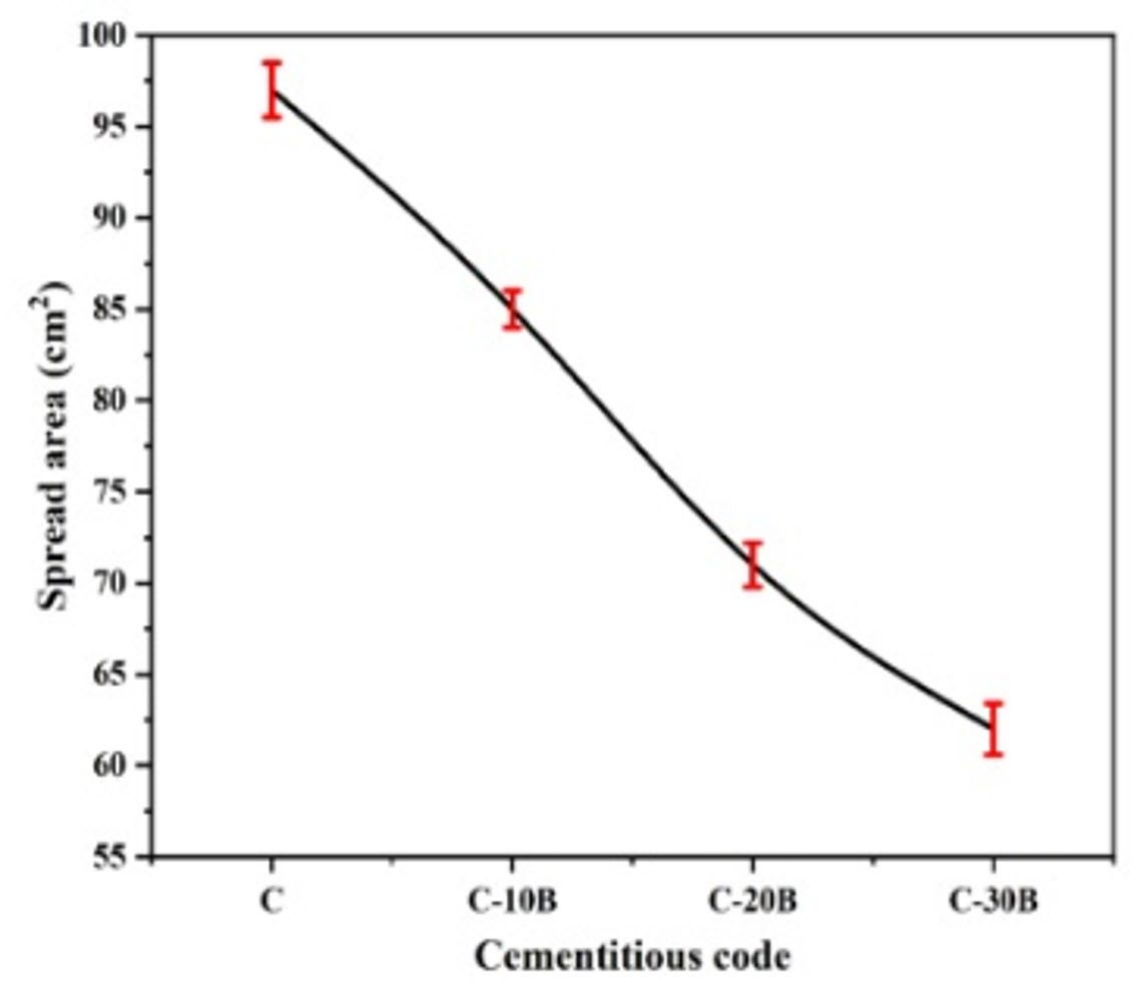
Workability of blended cement pastes containing different percentages of basalt.

The spread area of the prepared fresh cementitious composites (C, C-10B, C-20B, and C-30B) was measured, as demonstrated in Fig. 2, to evaluate the impact of different percentages of basalt on their workability. The spread area of the C specimen (100% OPC) is 97 cm^2^, while the basalt-containing composites are between 62 and 85 cm^2^. Partial replacement of OPC with 10, 20 and 30 wt% basalt reduces the spread area by 12.4, 26.8 and 36.1%, respectively, referring to the negative impact of basalt on the workability of fresh blended cement pastes, as observed by Laibao et al.^21^. They attributed the reduction in workability to the high surface-area of the basalt compared to OPC. As illustrated above, the surface area of OPC and basalt is 3320 and 4200 cm^2^/g, respectively. Generally, the high surface-area of mineral additives in the cement matrix adsorbs a large amount of mixing water, thus reducing workability^43,44^. Also, Akturk and Ayhan^45^ and Rashad et al.^19^ discussed the poor workability of basalt-containing cementitious materials to the morphology and surface texture characteristics of basalt. They reported that basalt grains have an angular shape and a rough surface, which increases friction between particles in the composite matrix, thus resisting the mixture’s rheology. Additionally, the filling effect of basalt causes the particles in the composite to aggregate, thereby limiting the space between them and preventing flowability^46^.

**Fig. 3:**
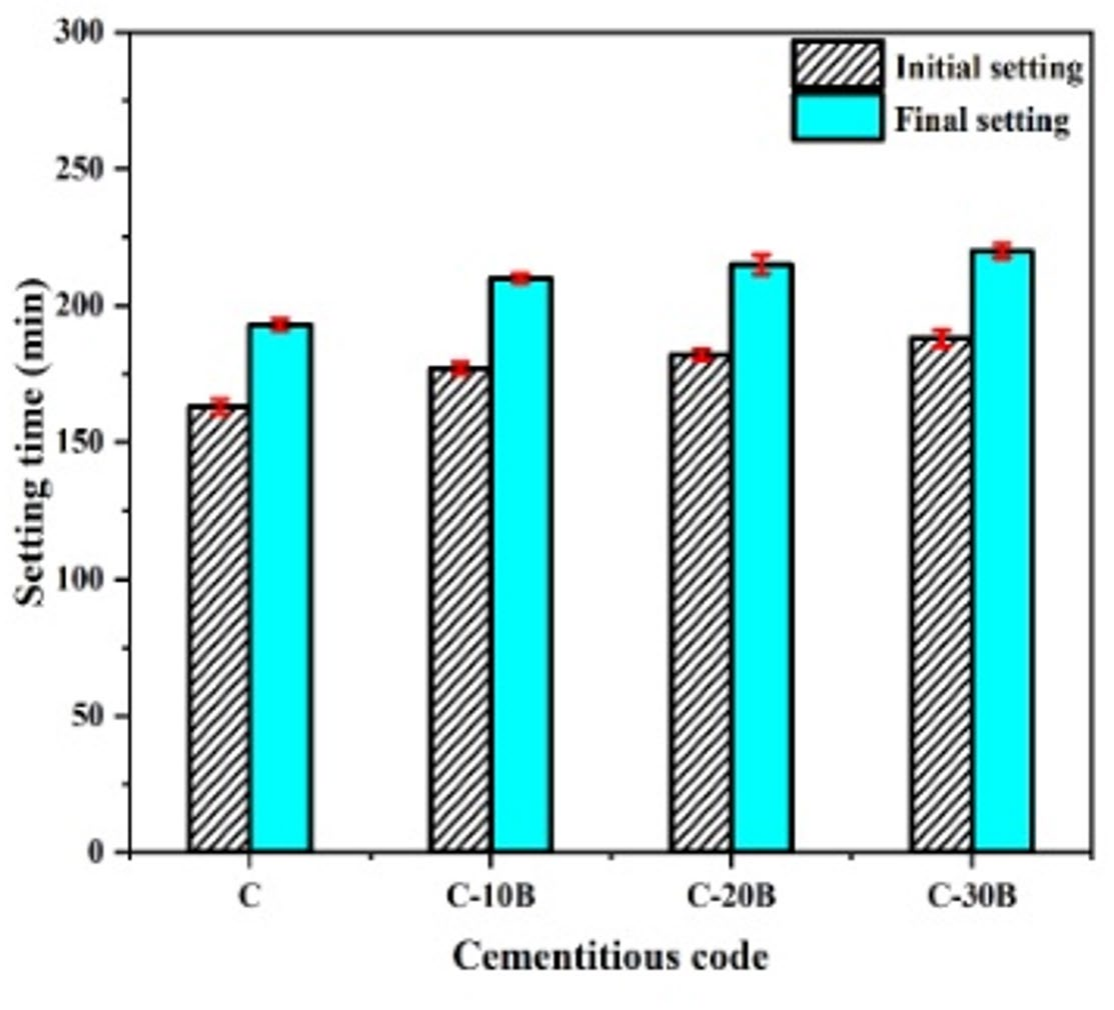
Setting time of blended cement pastes containing different percentages of basalt.

To investigate the effect of varying basalt percentages on the stiffening properties of blended cement pastes, the initial/final-setting time (I/F-ST) was measured, as in Fig. 3. It is detected that the IST values are 163, 177, 182 and 188 min and FST values are 193, 210, 215 and 220 min for C, C-10B, C-20B, and C-30B, respectively. These results clarify that basalt causes elongation in the I/F-ST of blended cement, matching with Dobiszewska and Beycioğlu^47^ and El-Didamony et al.^24^. Obviously, the I/F-ST values of specimens containing basalt are close to each other; a similar observation was detected by Laibao et al.^21^. The elongation mechanism of blended cement after incorporating basalt may be related to the dilution of cement, which causes (i) a reduction in the amount of celite (C_3_A) and alite (C_3_S) phases that are responsible for setting and early strength^21,24^; and (ii) decreasing the amount of portlandite phase (Ca(OH)_2_), which is required for the reaction with mineral admixture such as basalt^48^. Additionally, the low reactivity of basalt compared to OPC reduces the amount of hydration products formed, subsequently elongating the I/F-ST^45^.

### Compressive strength

**Fig. 4:**
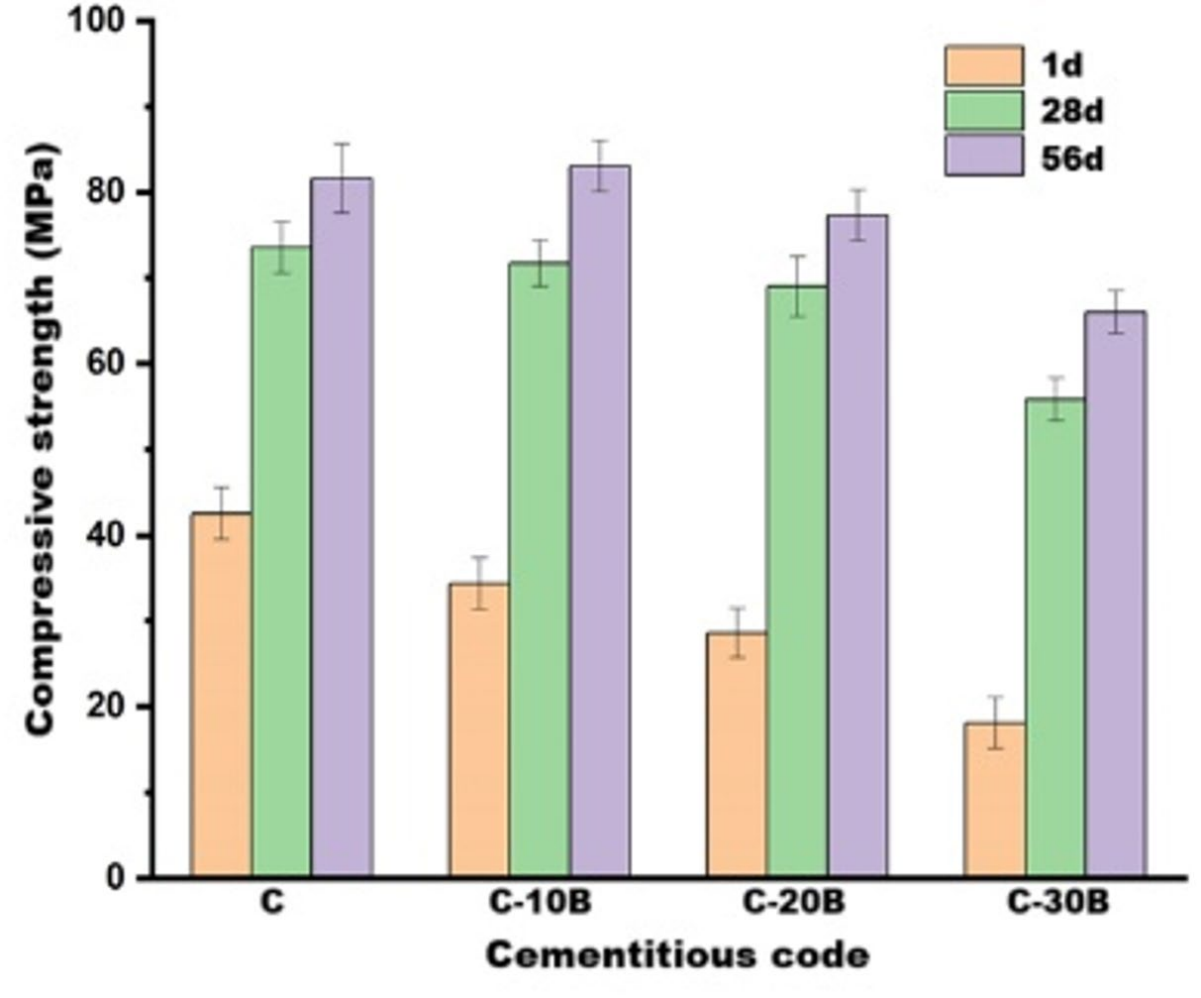
Compressive strength of normally cured blended cement pastes containing different percentages of basalt.

The impact of basalt content (0, 10, 20 and 30 wt% concerning OPC) on the compressive-strength of the normally cured OPC-basalt composites at 1, 28 and 56 days is represented in Fig. 4. Generally, it is detected that all fabricated composites exhibit significant improvement in the compressive-strength as the curing process continued^49,50^. This may result from ongoing advancements in the hydration process, which produce strength-giving-phases such as calcium-silicate-hydrate (CSH, tobermorite, jennite or amorphous gel), calcium-alumino-silicate-hydrate (CASH, stratlingite), and calcium-aluminate-hydrate (CAH, hydrocalumite and/or hydrogarnet). These phases form around cement grains, filling the open pores; thereby increasing the gel (strength-giving-phases)/pore ratio, which subsequently boosts mechanical performance^51,52^.

Additionally, Fig. 4. shows a reduction in compressive-strength values with increasing basalt content at early-ages; these values decreased by 19.1, 32.7 and 57.4% at 1-day for C-10B, C-20B and C-30B, respectively, compared with the C specimens. Conversely, at later-ages (28 and 56 days), the compressive-strength values of C-10B and C-20B specimens become close to control; the values are 73.5, 71.7 and 69 MPa at 28-days and 81.6, 83 and 77.3 MPa at 56-days for C, C-10B and C-20B, respectively. These results are in line with data obtained by Laibao et al.^21^ and Sevinç and Durgun^53^. At an early age, the reduction in strength values may be attributed to the dilution of cement with basalt, which has a relatively low pozzolanic activity^45^. Furthermore, cement dilution slows the rate of hydration^48^ and decreases the amount of the strength-giving-phases that are responsible for achieving mechanical performance^54^. Also, the decrease in cement content is accompanied by a reduction in calcium hydroxide (CH, portlandite), which is required for the pozzolanic reaction, resulting in a delay in the reaction of basalt^48^. At later ages, the relative enhancement in the strength of the basalt-blended composite may be due to its slight enhancement in the pozzolanic reaction, which results from its high specific surface-area, as well as its high SiO_2_ (45.97 wt%) and Al_2_O_3_ (14.61 wt%) content. Basalt can consume the CH produced by OPC hydration, causing the formation of additional hydration products that compensate for the decrease in their quantity resulting from cement dilution^48,53^. Besides the pozzolanic reaction, the presence of basalt in the matrix enhances the dispersion of cement grains^54^. The high specific surface-area also causes the basalt to act as a filler that densifies the microstructure^55^ and as nucleation sites that allow the hydration of unreacted particles^54^, thereby improving compressive-strength.

**Fig. 5:**
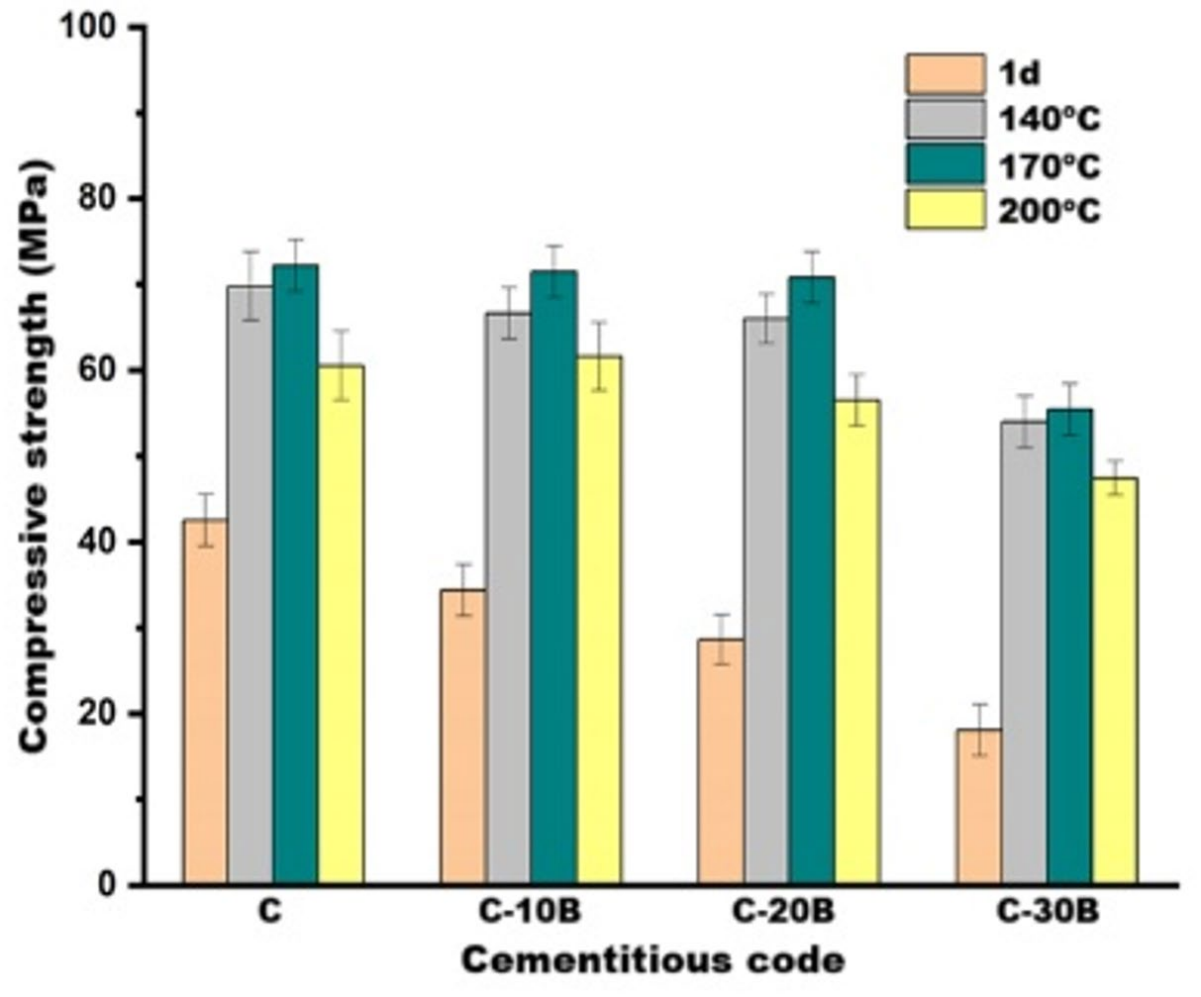
Compressive strength of hydrothermally cured blended cement pastes containing different percentages of basalt.

To investigate the ability in fabrication of pre-cast building materials from the developed composites (C, C-10B, C-20B and C-30B), the effect of hydrothermal curing conditions (140, 170 and 200 °C for 3 h) on their compressive-strength was studied as demonstrated in Fig. 5. Generally, it is observed that at all curing temperatures, C-10B and C-20B have compressive-strength close to C specimen, while C-30B is lower, due to the same reasons illustrated above. With increasing curing temperatures up to 170 °C, a continuous increase in the compressive strength is detected. At 170 °C/3 h, the compressive-strength values are 72.2, 71.5, 70.8 and 55.4 MPa, which are close to the values at 28-days (73.5, 71.7, 69 and 55.8 MPa) for C, C-10B, C-20B and C-30B, respectively. This means that the curing process at 170 °C for only 3 h is sufficient to achieve a compressive-strength comparable to that obtained through normal curing at 28-days, highlighting the significant impact of hydrothermal curing in reducing construction time. The improvement in compressive-strength with hydrothermal curing may be attributed to the internal autoclaving process, which accelerates the hydration of unreacted cement particles^56,57^. Moreover, autoclaving at an adequate temperature enhances the pozzolanic activity of basalt, facilitating a reaction with CH and producing additional strength-giving phases^56,58^. Li et al.^59^ and Shen et al.^60^ revealed that increasing the temperature during the hydration reaction enhances the pozzolanic activity of basalt, refining the pore-structure via forming extra hydration products. Additionally, hydrothermal curing leads to a redistribution of the pore structure, resulting in the formation of a denser matrix^61,62^. With the increasing hydrothermal curing temperature to 200 °C, the specimens show a loss in strength values of 16.2, 14.0, 20.2 and 14.4% for C, C-10B, C-20B, and C-30B, respectively, compared with other specimens cured at 170 °C. The extremely high pressure at 200 °C led to an increase in internal stress, forming cracks, thereby compromising the structural integrity^62^. These findings align with the research by Ramadan et al.^63^ and Amin et al.^64^.

Finally, it can be concluded that the incorporation of basaltic mineral phases, specifically anorthite, albite, augite and magnetite, into pozzolanic cementitious systems plays a crucial role in influencing hydration kinetics, nucleation dynamics, and the formation of secondary reaction products. Anorthite, characterized by its elevated calcium content, significantly contributes to late-stage hydration by releasing Ca^2+^ and Al^3+^ ions, which promote the development of calcium silicate hydrates such as tobermorite and jennite, as well as aluminous compounds including hydrocalumite (C_4_AH_13_ hydrogarnet (C_3_AH_6_), stratlingite (C_2_ASH_8_), and gehlenite-based hydrates. These hydration products are recognized for their contribution to mechanical strength. Albite, although less reactive due to its sodium-rich composition, supplies Al^3+^ that supports the formation of hydrogarnet and stratlingite, particularly under the high alkalinity conditions typical of pozzolanic environments. Augite, a multi-component silicate containing Ca, Mg, Fe, and Si, facilitates prolonged hydration and the generation of iron-aluminate phases such as xuite (Ca_3_Al_3_Fe_2_O_12_H_3_), which enhance matrix densification and long-term durability. Magnetite, while relatively inert during the initial hydration stages, acts as a nucleation site for iron-rich phases, including xuite. Under standard curing conditions, these basaltic minerals primarily function as nucleation centers and micro-fillers; however, under hydrothermal curing, their dissolution and mutual interactions create a chemically diverse environment that sustains hydration over extended periods, encourages the sequential formation of stable secondary phases mentioned above, and improves pore structure refinement. This collective behavior ultimately enhances the mechanical performance and durability of the cementitious matrix.

### Phase identification

#### X-ray diffraction analysis

**Fig. 6:**
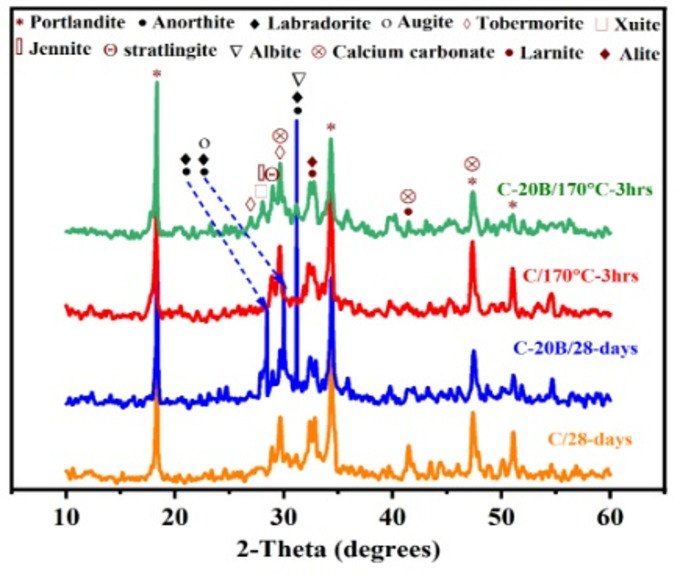
XRD patterns for some C and C-20B specimens at different curing regimes.

The XRD-pattern of the composite containing-basalt (C-20B, 80%OPC + 20%basalt) was compared with that of the control specimen (C, 100%OPC) to identify the effect of basalt on the phase composition of the blended cement at 28-days, as shown in Fig. 6. The XRD-pattern of the C specimen shows the presence of distinguishable crystalline phases related to formed hydration products and some remaining unreacted phases. The hydration products formed are (i) portlandite (CH, Ca(OH)_2_, PDF# 96-900-0114) at 2*θ* = 18.35, 34.29, 47.43 and 51.09°; (ii) stratlingite (CASH, Ca_2_Al_2.11_Si_1.11_O_16.25_H_18_, PDF# 96-900-5060) at 2*θ* = 29.01° and tobermorite (CSH, Ca_2_Si_3_O_11_H_6_, PDF# 96-900-2247) at 2*θ* = 29.61°, which are considered the main strength-giving-phases responsible for gaining strength; and (iv) calcite (CC, CaCO_3_, PDF# 96-101-096) at 2*θ* = 29.61 and 47.43°, which is resulted from the carbonation of CH by CO_2_ from atmosphere during handling. The peak of the unreacted phases are larnite (β-C_2_S, Ca_2_SiO_4_, PDF# 96-901-2790) appeared at 2*θ* = 32.49 and 41.53° and alite (C_3_S, Ca_3_SiO_5_, PDF# 96-154-0706) at 2*θ* = 32.49°. The same phases are identified by Mohsen et al.^12,44^ and Habib et al.^65^. After incorporating basalt into the cement matrix (C-20B), the same phase was identified, with some minor changes, such as (i) diminishing the intensities of the peaks of unreacted phases (β-C_2_S and C_3_S) and hydration products (CH, CASH, CSH, and CC) due to the dilution of the cement with 20 wt% basalt, align with Laibao et al.^21^ and Mohsen et al.^44^; and (ii) appearance of new peaks related to the unreacted phases of basalt, such as anorthite (CaAl_2_Si_2_O_8_, PDF# 96-100-0035) and labradorite (Ca_0.67_Al_1.67_Si_2.33_Na_0.33_O_8_, PDF# 96-210-8234) at 2*θ* = 28.45, 30.01 and 31.19°, as well as augite (Ca_0.6_Al_0.34_Si_1.82_Fe_0.2_Mg_0.9_Na_0.1_O_6_, PDF# 96-100-0036) at 2*θ* = 30.01° and albite (AlSi_3_NaO_8_, PDF# 96-900-3703) at 2*θ* = 31.19°. The same observation was detected by Rashad et al.^19^. The existence of these phases refers to the weak pozzolanic activity of the basalt. This is strong evidence for the reason for the low compressive-strength of C-20B with respect to the C specimen at 28-days (69 and 73.5 MPa, respectively).

Additionally, Fig. 6 illustrates the effect of hydrothermal curing at 170 °C for 3 h on the phase composition of C and C-20B specimens. For the C/170°C–3 h specimen, it exhibits the same peaks observed in C/28-days, with comparable intensities. This indicates that hydrothermal curing at 170 °C for 3 h is sufficient to produce a quantity of hydration products similar to those obtained through normal curing for 28-days, as evidenced by the approximately equal compressive strength values of C/28-days and C/170°C–3 h (73.5 and 72.2 MPa, respectively). Regarding C-20B/170°C–3 h, it is clear that the peaks associated with the unreacted phases of basalt (anorthite, labradorite, augite, and albite) have disappeared, indicating that curing at higher temperatures and pressures has a high efficiency in improving the pozzolanic activity of basalt, which matches Saraya and El-Fadaly^66^. At the same time, new peaks allied to strength-giving-phases are detected at 2Ɵ= 26.93°, which is related to the tobermorite phase and at 2*θ* = 28.05°, affiliated with xuite phase (Ca_3_Al_3_Fe_2_O_12_H_3_, PDF# 00-073-0959) and jennite phase (Ca_9_Si_6_O_32_H_22_, PDF# 96-901-2922). This also discusses the closing of the compressive-strength values between C-20B/28-days and C-20B/170°C–3 h, which are 69 and 70.8, respectively.

#### Thermal analysis

**Fig. 7:**
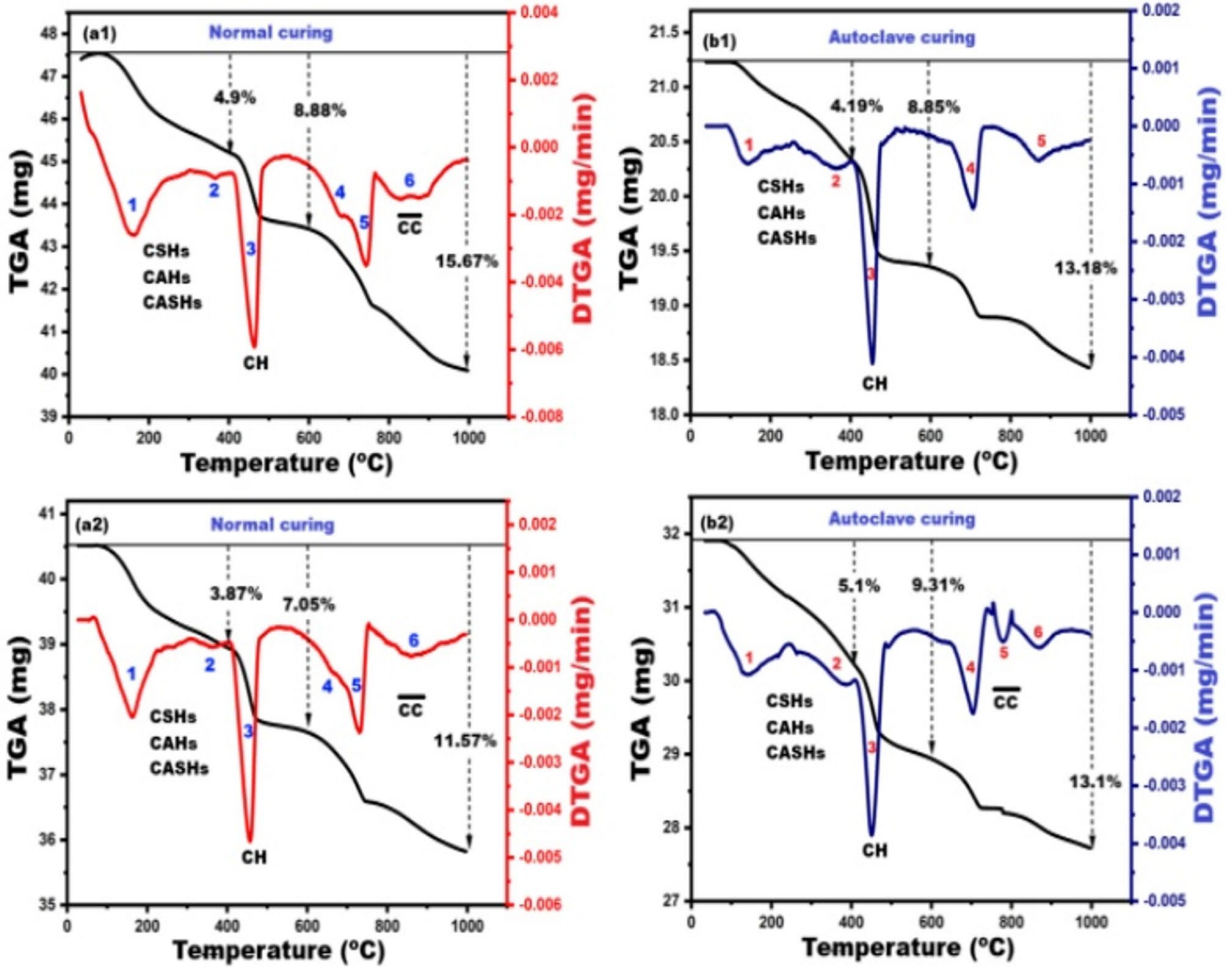
TGA/DTGA thermograms of: C pastes (**a1**,**b1**) and C-20B pastes (**a2**,**b2**) under different curing regimes.

Figure 7 presents the TGA/DTGA thermograms of the reference sample (C, 100% OPC) and the modified sample (C-20B, 80% OPC + 20% basalt powder), highlighting the mineralogical phase composition under different curing conditions. Figure 7 (a1, a2) displays the thermograms for normally cured pastes up to 28 days, while Fig. 7b1,b2 shows the results for samples subjected to autoclaving at 170 °C for 3 h. In Fig. 7a1, thermal analysis of sample C up to 1000 °C under a nitrogen atmosphere reveals four distinct mass loss events: First mass loss (50–400 °C): A 4.9% weight loss is observed, accompanied by two endothermic peaks (1 and 2). This is attributed to the dehydration of various crystalline and amorphous calcium silicate hydrates (CSHs), including tobermorite, jennite, and CSH gel^44,61,67–69^, in addition to dehydration of hydrocalumite (C₄AH₁₃) ^70^, hydrogarnet (C₃AH₆)^7,29^, gehlenite hydrates (2CaO.Al_2_O_3_.SiO_2_·nH₂O)^71^ and stratlingite (C₂ASH₈). These phases are known to contribute significantly to mechanical strength. Second mass loss (400–600 °C): A 3.76% weight loss occurs, marked by a prominent endothermic peak (3), corresponding to the dehydroxylation of portlandite (Ca(OH)₂)^72^.Third and fourth mass losses (600–1000 °C): A combined 7.01% weight loss is recorded, with three endothermic peaks (4, 5, and 6) indicating the decomposition of calcium carbonate polymorphs as vaterite, aragonite, and calcite^50,73^.

The partial replacement of OPC with 20% basalt powder significantly influenced the thermal decomposition behavior of the paste, as illustrated in Fig. 7a2. The mass loss percentages within the temperature ranges of 50–400 °C, 400–600 °C, and 600–1000 °C were reduced to 3.87%, 3.18%, and 4.52%, respectively. These reductions indicate a lower formation of hydration products (CSHs, CAHs, CASHs), which are primarily responsible for mechanical strength development. As a result, the compressive strength of the modified mixture (C-20B) decreased slightly from 73.5 MPa to 69 MPa after 28 days of standard curing. Despite this reduction, the strength remained relatively high, suggesting that the pozzolanic activity^19^ of basalt powder contributed positively to the microstructure. This is further supported by the observed decrease in Ca(OH)_2_ content, from 3.76% in the control mixture to 3.18% in the modified blend. Additionally, the data indicates a lower degree of carbonation in the C-20B mixture compared to the control, implying enhanced durability characteristics.

Figure 7b1,b2 illustrates the TGA/DTGA thermograms of the C and C-20B pastes subjected to hydrothermal curing at 170 °C/3 h. This short-duration treatment under steam pressure was sufficient to generate hydration products comparable in both type and quantity to those formed under standard curing conditions at 28 days. Consequently, the mechanical performance of the hydrothermally cured samples was nearly identical to that of the conventionally cured counterparts, indicating the effectiveness of accelerated curing in achieving similar microstructural development^74^.

### Morphology

**Fig. 8:**
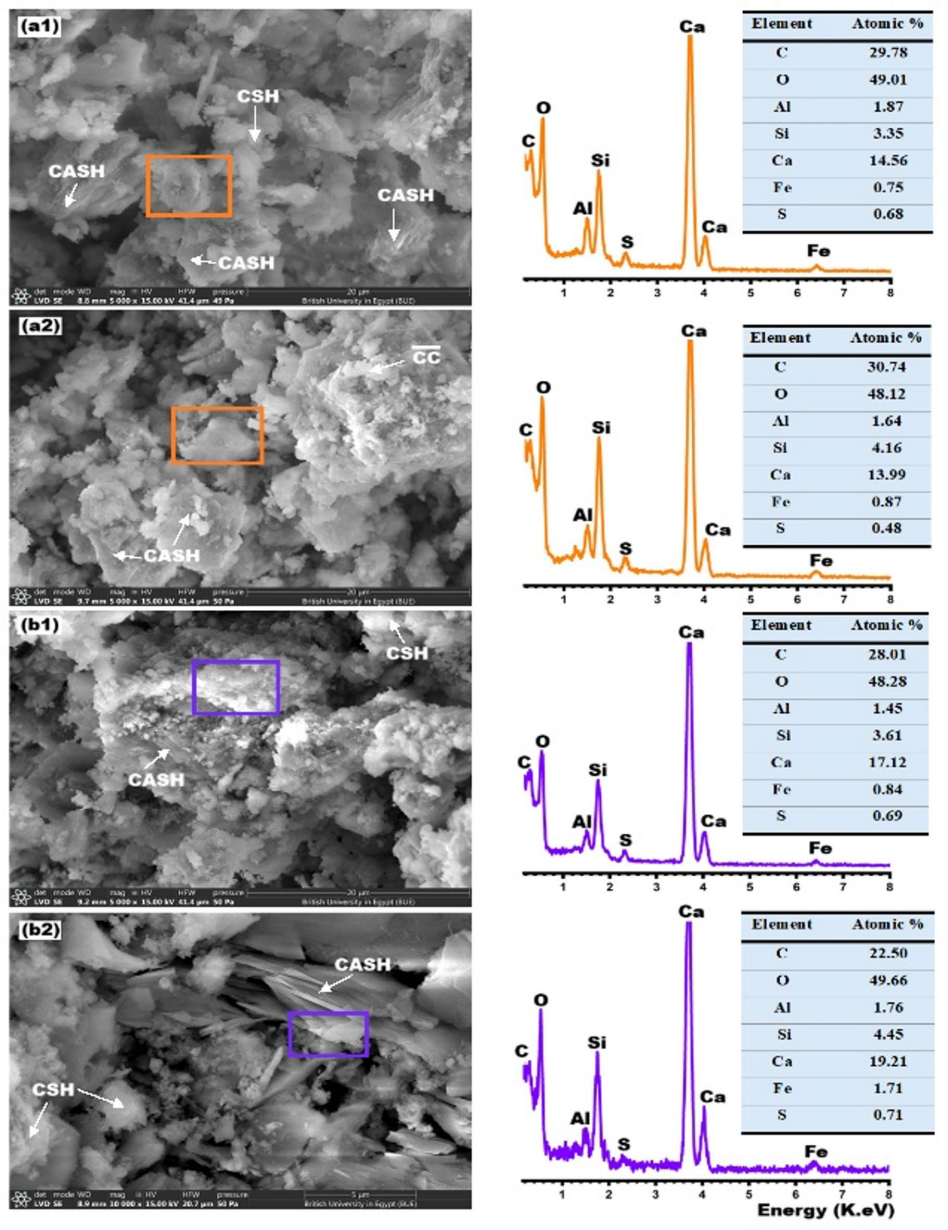
SEM/EDX images of: (**a1**) C/28d, (**a2**) C-20B/28d, (**b1**) C/170 °C, (**b2**) C-20B/170 °C.

The geometric morphology of hydrates (CSHs, CASHs, CAHs, CC and CH) formed during cementitious reactions plays a crucial role in determining the mechanical properties and durability of concrete^75–77^. Figure 8 displays SEM/EDX images for C and C-20B pastes at various curing regimes. Figure 8a1 shows an SEM/EDX image of cement paste (C) at 28-days of curing. Irregular and dense structures of CSH gel interweaved with layered sheets of CASHs^13^. CSH gels significantly improve the cohesion between cement particles while simultaneously reducing the overall porosity of the matrix^12^, thereby enhancing the composite’s resistance to aggressive environmental conditions^51^. In addition, the presence of irregularly shaped silicate hydrates contributes to the effective filling of micro-voids and capillary pores, leading to a more refined pore structure and decreased permeability^78^. Partial replacement of OPC with 20% basalt powder (Fig. 8a2) leads to the formation of additional stacked plate-like CASH crystals^71^, which contribute to the densification and reinforcement of the C-20B microstructure. EDX analysis indicates that the Si/Al ratio increases from 1.87 in the control paste to 2.65 in the C-20B paste, while the Ca/Si ratio decreases from 6.21 to 4.80, respectively. These findings suggest that the reference paste is richer in CSH gels^79^, whereas the C-20B paste contains a higher concentration of CASH phases due to the pozzolanic capability of basaltic binders^80^. Figure 8b1,b2 illustrates the influence of hydrothermal treatment at 170 °C/3hrs on the morphological characteristics of the C and C-20B samples. Under steam pressure, highly crystalline phases such as stratlingite and tobermorite are formed^81^. EDX analysis indicates that the Si/Al ratio increases from 1.79 in the control paste to 2.53 in the C-20B paste, while the Ca/Si ratio decreases from 4.34 to 3.36, respectively. These findings suggest that the reference paste is richer in CSH gels^79^, whereas the C-20B paste contains a higher concentration of CASH phases due to the pozzolanic capability of basaltic binders^80^. Figure 8b1,b2 illustrates the influence of hydrothermal treatment at 170 °C/3 h on the morphological characteristics of the C and C-20B samples. Under steam pressure, highly crystalline phases such as stratlingite and tobermorite are formed^81^. EDX elemental analysis reveals that the Ca/Si ratio in the control sample increases from 4.34 to 4.74, while the Si/Al ratio rises from 1.79 to 2.48. Similarly, in the C-20B sample, the Ca/Si ratio increases from 3.36 to 4.31. These increases reflect phase transformations associated with a high degree of crystallinity^58,82,83^, which in turn enhances the densification and structural reinforcement of the C and C-20B matrices.

### Shielding and pore structure analysis

Shielding against gamma rays is a critical application across various fields, including nuclear power plants, medical imaging facilities and radioactive waste storage^84^. The effectiveness of cementitious composites in blocking gamma rays largely depends on their composition, incorporating specific additives, curing regimes, pore structure and the operating temperature^58,75^.

**Fig. 9:**
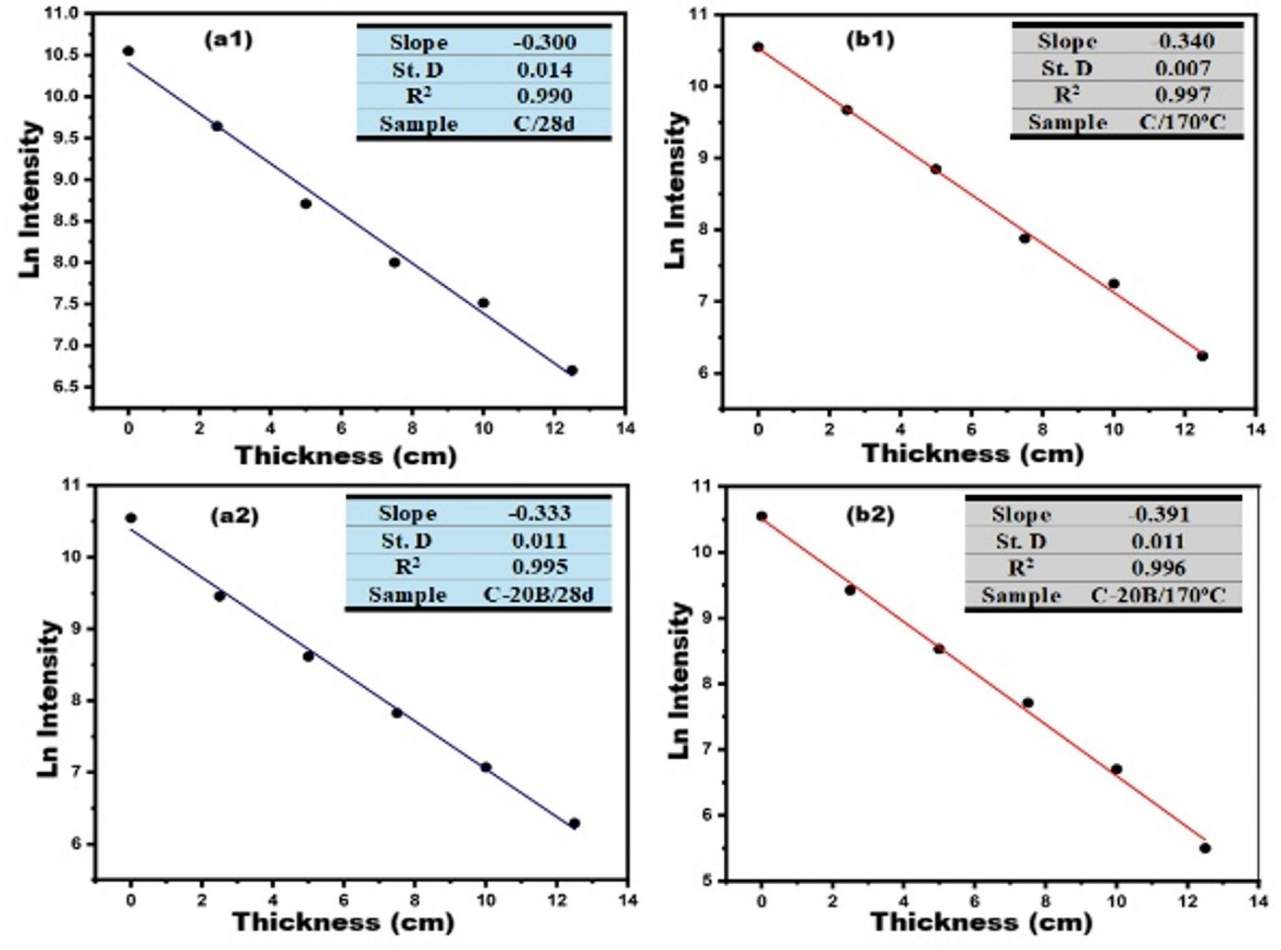
Relation between intensity of transmitted gamma ray and thickness of different pastes: (**a1**) C/28d, (**a2**) C-20B/28d, (**b1**) C/170 °C, (**b2**) C-20B/170 °C.

**Fig. 10:**
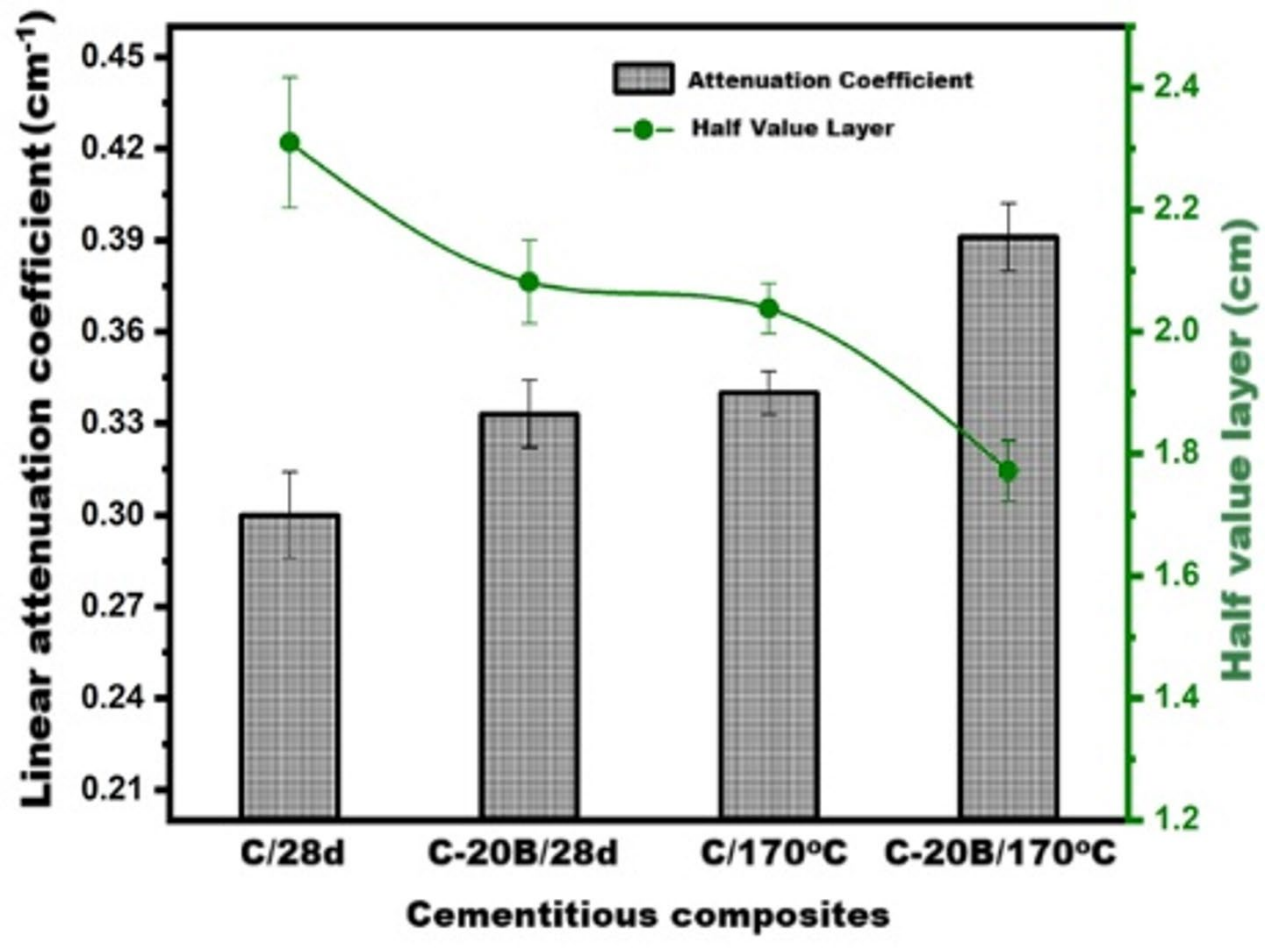
Values of attenuation coefficients and HVL of C and C-20B pastes under normal and hydrothermal curing.

Figure 9 illustrates the correlation between the transmitted intensity of gamma rays (emitted from ^137^Cs-isotope at 661.64 keV) and the thickness of cementitious pastes composed of ordinary Portland cement (OPC) and OPC partially replaced with 20% basalt, subjected to two distinct curing regimes. All investigated samples (C/28d, C-20B/28d, C/170 °C, and C-20B/170 °C) exhibited strong adherence to the Beer–Lambert law, with correlation coefficients (R^2^) ranging from 0.990 to 0.997. A progressive increase in sample thickness up to 12.5 cm resulted in a pronounced reduction in the transmitted gamma-ray intensity. Under standard curing conditions (immersion in tap water at 25 °C for 28 days), the linear attenuation coefficients (µ) for the C and C-20B samples were determined to be 0.300 ± 0.014 cm^−1^ and 0.333 ± 0.011 cm^−1^, respectively (Fig. 9a1,a2). Transitioning to hydrothermal curing at 170 °C for 3 h under autoclave steam pressure led to a notable increase in µ values, reaching 0.340 ± 0.007 cm^−1^ for C and 0.391 ± 0.011 cm^−1^ for C-20B (Fig. 9b1,b2). Correspondingly, the half-value layer (HVL), as shown in Fig. 10, decreased with both basalt incorporation and hydrothermal treatment: 2.31 ± 0.107 cm (C/28d), 2.08 ± 0.068 cm (C-20B/28d), 2.038 ± 0.041 cm (C/170 °C), and 1.772 ± 0.049 cm (C-20B/170 °C). These findings underscore the significant influence of both the binder composition and curing method on the gamma-ray attenuation performance. The partial replacement of OPC with 20% basalt enhanced the shielding efficiency by approximately 10% under normal curing and by about 30% under hydrothermal conditions. This improvement is attributed to the presence of radiation-absorbing mineral phases in basalt, such as anorthite, albite^85^, labradorite^86^, augite^87^, and magnetite, which function as effective dosimetric structures^88^. Additionally, the pozzolanic reactivity of basalt promotes the formation of secondary phases, including xuite, jennite, tobermorite, and strätlingite, which contribute to microstructural densification^89^ and further enhance the radiation shielding capacity of the C-20B pastes.

**Fig. 11:**
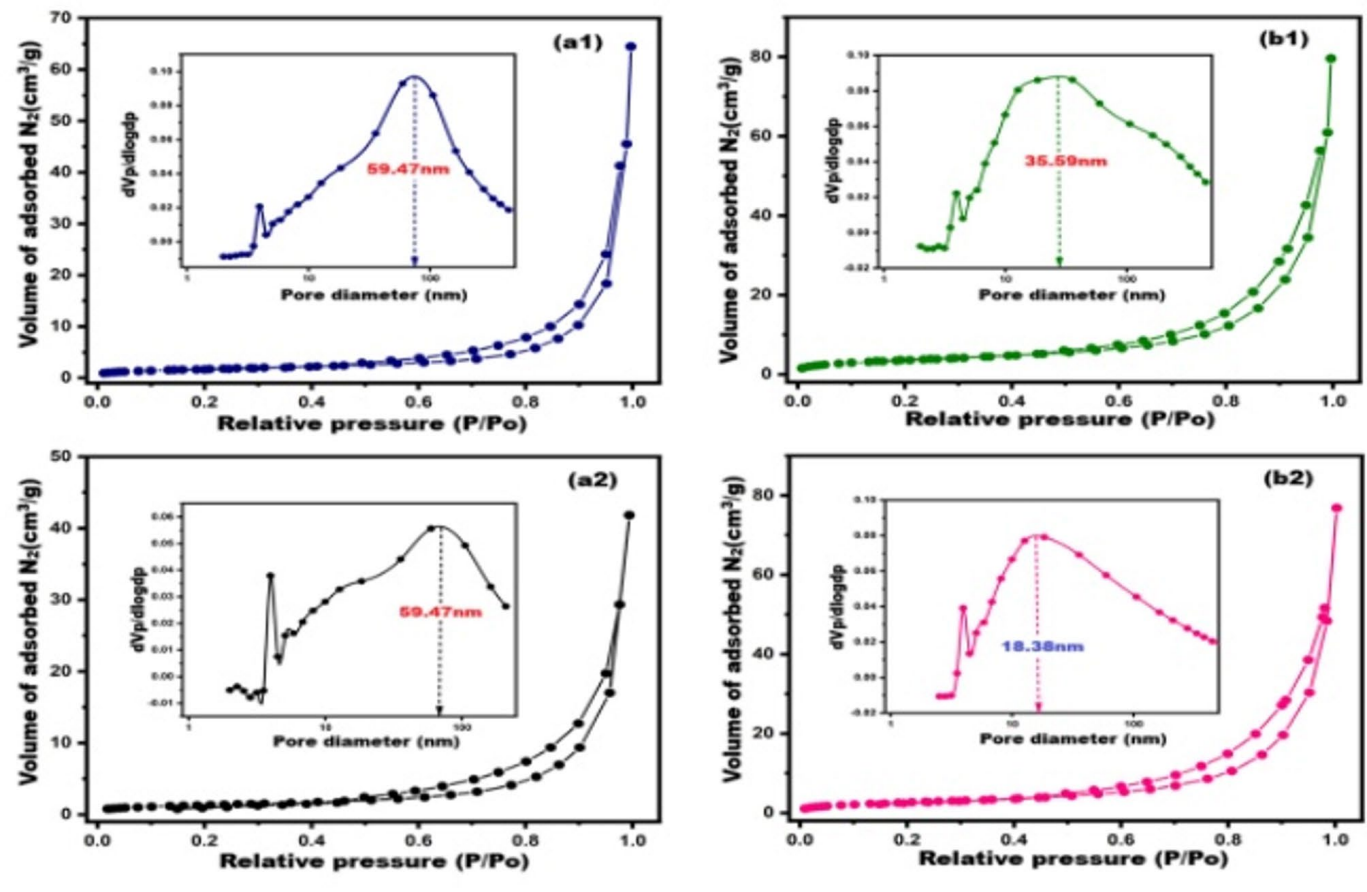
N_2_-adsorption/desorption isotherm/pore size distribution of: (**a1**) C/28d, (**a2**) C-20B/28d, (**b1**) C/170 °C, (**b2**) C-20B/170 °C.

Investigating the porous structure of cementitious materials provides valuable insights into their radiation shielding performance. Figure 11 presents the N_2_ adsorption–desorption isotherms and corresponding pore size distribution curves for C/28d, C-20B/28d, C/170 °C, and C-20B/170 °C, analyzed using the BET and BJH models. All samples exhibit Type III isotherms with H3 hysteresis loops, characteristic of mesoporous structures typically found in cement-based materials^64,77,90^. The BJH pore size distribution analysis reveals that the maximum pore diameters (dpmax) for C/28d, C-20B/28d, C/170 °C, and C-20B/170 °C are 59.47 nm, 59.47 nm, 35.39 nm, and 18.38 nm, respectively. Additionally, as summarized in Table 3, the average pore diameters (APD) are 42.82 nm, 26.38 nm, 27.31 nm, and 23.59 nm, respectively. These results demonstrate that hydrothermal treatment at 170 °C for just 3 h significantly refines the pore structure, leading to a denser and more compact microstructure. This densification is likely to enhance the material’s ability to attenuate gamma radiation^51,61,78,91,92^. Notably, the C-20B/170 °C sample exhibits the most refined porous structure, with the smallest dpmax and APD values, and concurrently achieves the highest linear attenuation coefficient (0.391 ± 0.011 cm^−1^). This corresponds to a half-value layer (HVL) of 1.772 ± 0.049 cm, indicating that only this thickness is required to reduce the intensity of 661.64 keV gamma rays by 50%.

**Table 3:** Pore structure parameters for C and C-20B at different curing regimes.

Cementitious paste	S.S.A (m^2^/g)	Vp (cm^3^/g)	dpmax (nm)	APD (nm)
C/28d	6.23	0.074	59.47	42.82
C-20B/28d	4.73	0.060	59.47	26.38
C/170 °C	13.06	0.094	35.95	27.31
C-20B/170 °C	9.84	0.087	18.38	23.59

### Comparison with previous studies

This section presents a comparison of the roles played by basalt, various artificial pozzolans, certain volcanic pozzolans, and other conventional radiation shielding materials in terms of their mechanical properties, hydration behavior, and shielding capabilities.

#### Mechanical performance with other artificial Pozzolana

The mechanical behavior of basalt-blended cement has attracted increasing interest as a sustainable alternative to conventional Portland cement systems. This section offers a comparative evaluation of basalt-based mixtures alongside other supplementary cementitious materials (SCMs), including fly ash (FA), silica fume (SF), and ground granulated blast furnace slag (GGBFS), under both standard and hydrothermal curing conditions. Basalt, commonly used as a filler, exhibits limited pozzolanic activity during early hydration stages. However, its performance improves notably over time due to progressive pozzolanic reactions. In our investigation, a 20% replacement of Portland cement with basalt demonstrated optimal outcomes in terms of compressive strength and microstructural densification. Under hydrothermal curing, basalt’s reactivity was significantly enhanced, promoting the formation of beneficial hydration products such as tobermorite, jennite, hydrocalumite, hydrogarnet, stratlingite, and calcium-iron-aluminate phases like xuite. Fly ash is characterized by low early-age strength due to its slower pozzolanic reaction kinetics, yet it contributes substantially to long-term strength and durability. Silica fume, on the other hand, possesses high pozzolanic reactivity, leading to rapid strength development and reduced porosity, making it particularly suitable for high-performance concrete applications. Hydrothermal curing accelerates both hydration and pozzolanic reactions across all SCMs. Under these conditions, basalt exhibits a marked increase in reactivity, thereby narrowing the performance gap between itself and more reactive materials such as silica fume and slag. Table 4 clarifies a comparative mechanical performance for different pozzolanic cements containing basalt, fly ash, slag, and silica fume.

**Table 4:** Comparative mechanical performance of some artificial pozzolana-containing blended cements.

Type of composite paste	Replacement (%)	Compressive strength (MPa)			References
		Early age (1–7 days)	Later age 28 days	Hydrothermal curing	
OPC	–	42.5	73.5	72 MPa at 170 °C/3 h	This study
OPC-Basalt	10–20%	28–34	69–72	70–72 MPa 170 °C/3 h	This study
OPC-fly ash	20%	30–40	65–75	70-80 MPa 90 °C/48 h	^93–96^
OPC-Slag	20–80%	35–55	50–90	73-96 MPa 45–85 °C/60 days	^97–101^
OPC-Silica fume	5–15%	40–50	70–80	60 °C/7 days	^102–104^

#### Hydration kinetic with other volcanic Pozzolana

The effectiveness of volcanic pozzolanic materials such as basalt powder, pumice, tuff, and scoria in modifying cement hydration kinetics is governed by their particle size, amorphous content, and chemical composition. Finer particles offer increased surface area, which enhances the rate of hydration reactions. This effect is particularly pronounced in pumice and tuff, which show significant improvements in reactivity when finely ground. The presence of high amorphous silica and alumina content is a key factor in pozzolanic performance, with pumice and tuff ranking highest in this regard^105,106^. Furthermore, the combined content of reactive oxides (SiO_2_, Al_2_O_3_, and Fe_2_O_3_) is critical to the material’s chemical reactivity. Pumice and tuff are notably rich in these oxides, whereas basalt contains them in moderate amounts^107^, and scoria typically has lower levels^108^. These characteristics collectively determine the extent and timing of hydration reactions, influencing both early and long-term performance in cementitious systems as displayed in Table 5.

**Table 5:** Comparative influence of volcanic pozzolans on cement hydration kinetics.

Volcanic pozzolana	Hydration kinetic		Key factors	Reference
	Early age	Later age		
Basalt	Hydration reaction is slow due to limited pozzolanicity	Moderate	Coarse particles, low amorphous content and moderate content of reactive SiO_2_, Al_2_O_3_, and Fe_2_O_3_	This study
Pumice	Hydration reaction is fast due to strong pozzolanicity	Fast	Fine particles, high amorphous content and high content of reactive oxides	^105^
Tuff	Moderate hydration	Fast	Volcanic glass content, moderate fineness, reactive composition	^106^
Scoria	Very slow hydration	Slow	Coarse texture, low amorphous content, moderate content of reactive oxides	^108^

#### Shielding capability with other traditional shielding materials

The integration of basalt powder into cementitious composites, particularly when combined with hydrothermal treatment, significantly enhances their ability to attenuate gamma radiation. Basalt’s dense mineral composition, rich in iron, magnesium, and calcium silicates, contributes to increased bulk density and photon interaction, which are essential for effective radiation shielding^109^. Hydrothermal treatment at 170 °C/3 h further improves shielding performance due to the presence of radiation-absorbing mineral phases in basalt, such as anorthite, albite^85^, labradorite^86^, augite^87^, and magnetite, which function as effective dosimetric structures^88^. Additionally, the pozzolanic reactivity of basalt promotes the formation of secondary phases, including xuite, jennite, tobermorite, and stratlingite, which contribute to microstructural densification^89^ and further enhance the radiation shielding capacity of OPC-20%basalt, thereby increasing the linear attenuation coefficient (µ) and reducing the half-value layer (HVL). Compared to traditional radiation-shielding concretes, basalt-based systems offer a promising alternative. Barite concrete, with a density of 3.5–4.0 g/cm^2^, exhibits the highest attenuation capacity (HVL ~ 2.1–3.0 cm), followed by hematite and magnetite concretes, which also provide strong shielding due to their high iron content and densities around 3.2–3.6 g/cm^2^^110^. Basalt composites, especially when hydrothermally treated, achieve densities of approximately 2.9–3.0 g/cm^2^ and HVL values around 1.77 cm, making them suitable for shielding applications. Basalt-based composites present a cost-effective and environmentally sustainable option for general-purpose radiation shielding in medical, industrial, and infrastructure contexts. Table 6 demonstrates shielding, economic, and logistical characteristics of some selected radiation-shielding materials.

**Table 6:** Comparative physical, shielding, economic, and logistical characteristics of selected radiation-shielding materials.

Shielding material	Density (g/cm^3^)	Linear attenuation coefficient (µ)	Cost-effectiveness	Availability	References
Barite	3.5–4.0	High µ ≈ 0.28–0.30 cm⁻¹	Moderate to high cost	Regionally variable; often imported	^111^
Magnetite	3.2–3.6	High µ ≈ 0.28–0.30 cm⁻¹	More cost-effective than barite	Widely available; industrial byproduct	^112^
Hematite	3.3–3.5	Moderate µ ≈ 0.18–0.21 cm⁻¹	Moderate cost	Readily available in many regions	^113,114^
Basalt treated at 170 °C/3 h	2.9–3.0	High µ = 0.39 cm⁻¹	Low-cost	Abundant and locally sourced in volcanic regions	This study

## Conclusions

This study presents a novel approach to utilizing basalt powder as SCM for developing pozzolanic cement composites with enhanced mechanical performance and radiation shielding capabilities. By partially replacing ordinary Portland cement (OPC) with basalt at varying levels (0, 10, 20, and 30 wt%), the research demonstrates the feasibility of producing eco-efficient and multifunctional cementitious materials suitable for precast applications. Based on the experimental results, the following conclusions can be drawn:

- The incorporation of basalt powder into OPC mixtures reduces the workability and prolongs the setting time of fresh pastes.
- Although early-age compressive strength is adversely affected by basalt addition, significant strength gains are observed at later curing stages.
- Hydrothermal curing at 170 °C for 3 h is sufficient to achieve compressive strength comparable to that obtained through 28 days of ambient curing.
- A mix containing 20 wt% basalt was identified as the optimal formulation, offering a favorable balance of mechanical performance, economic viability, and environmental sustainability.
- Hydrothermal treatment promotes the formation of strength-contributing phases such as tobermorite, jennite, xuite, hydrocalumite, hydrogarnet, strätlingite, and gehlenite hydrates, as confirmed by XRD, TGA/DTGA, and SEM/EDX analyses.
- The mesoporous structure of the cementitious matrix is developed by hydrothermal curing, as evidenced by BET/BJH analysis.
- The partial replacement of OPC with 20 wt% basalt improves gamma radiation shielding efficiency by approximately 10% under ambient curing and by about 30% under hydrothermal conditions.

## Data Availability

All data generated or analyzed during this study are included in this published article.
